# Associations between neurodevelopmental traits and perinatal depression: a prospective study in Japanese women

**DOI:** 10.1007/s00737-026-01781-4

**Published:** 2026-11-04

**Authors:** Kyoko Hasegawa, Daimei Sasayama, Hideo Honda, Norihiko Kikuchi, Takaaki Hondo, Kenji Itakura, Tomoko Watanabe, Shinsuke Washizuka

**Affiliations:** 1 Department of Pediatrics, Minami-Nagano Medical Center, Shinonoi General Hospital, Nagano, Japan; 2 Graduate School of Medicine, Science and Technology, Shinshu University, Matsumoto, Japan; 3 Mental Health Clinic for Children, Shinshu University Hospital, Matsumoto, Japan; 4 Department of Child and Adolescent Developmental Psychiatry, Shinshu University School of Medicine, Matsumoto, Japan; 5 Department of Psychiatry, Shinshu University School of Medicine, Matsumoto, Japan; 6 Department of Obstetrics and Gynecology, Shinshu University School of Medicine, Matsumoto, Japan; 7 Present Address: Department of Obstetrics and Gynecology, Nagano Red Cross Hospital, Nagano, Japan; 8 Department of Obstetrics and Gynecology, Minami-Nagano Medical Center, Shinonoi General Hospital, Nagano, Japan; 9 Itakura Ladies Clinic, Nagano, Japan; 10 Maruyama Obstetrics and Gynecology Hospital, Nagano, Japan

**Keywords:** Perinatal depression, Perinatal mental health, ADHD traits, ASD traits, Neurodevelopmental disorder, EPDS

## Abstract

**Purpose:**

This study examined how attention-deficit/hyperactivity disorder (ADHD) and autism spectrum disorder (ASD) traits relate to depressive symptoms from late pregnancy through early postpartum in Japanese women, alongside perinatal factors.

**Methods:**

In a multicenter prospective study, 1,104 women completed questionnaires at late pregnancy (T1), 2 weeks postpartum (T2), and 1 month postpartum (T3). Depressive symptoms were assessed using the Edinburgh Postnatal Depression Scale (EPDS), ADHD traits with the Adult ADHD Self-Report Scale (ASRS), and ASD traits with the Autism Spectrum Quotient (AQ). Chi-square tests evaluated timepoint-specific associations, multivariable logistic regression identified independent predictors of EPDS positivity at T2, and a linear mixed-effects model assessed longitudinal changes.

**Results:**

EPDS scores and EPDS positivity were highest at T1 and declined postpartum (16.7%, 8.6%, 4.0%). Scores were correlated across timepoints. In univariate analyses, antenatal EPDS positivity showed associations with ASD traits (AQ+: OR = 6.08, 95% CI 2.17–16.99) and ADHD traits (ASRS+: OR = 4.86, 95% CI 3.06–7.72). In multivariable analysis, independent predictors of EPDS positivity at T2 included ADHD traits (OR = 1.37, 95% CI 1.13–1.66), primiparity, psychiatric treatment history, and antenatal EPDS scores. ASD traits were not associated with T2 EPDS positivity after adjustment for antenatal EPDS scores. In the mixed-effects model, both ADHD and ASD traits were associated with higher EPDS scores during pregnancy, with attenuated effects postpartum.

**Conclusion:**

Neurodevelopmental traits are associated with perinatal mental health, and trait- and timing-informed support may enhance maternal psychological stability and caregiving outcomes.

**Supplementary Information:**

The online version contains supplementary material available at https://doi.org/10.1007/s00737-026-01781-4.

## Article Highlights

ADHD traits were associated with depressive symptoms from pregnancy through postpartum.

ASD traits were associated with depressive symptoms mainly during pregnancy.

Risk factors for postpartum depression include first-time mothers, psychiatric history, elevated antenatal EPDS scores.

Many neurodevelopmental traits remain unrecognized in clinical settings.

Personalized support based on trait profile and timing can improve maternal mental health.

**Supplementary Information:**

The online version contains supplementary material available at https://doi.org/10.1007/s00737-026-01781-4.

## Introduction

Perinatal depression is a major mental health concern that affects approximately 10 − 20% of women worldwide (Bauman et al. 2020; Tokumitsu et al. 2020; Wang et al. 2021). It is associated with a range of adverse outcomes, including impaired bonding, increased maltreatment risk, and later child difficulties (Dubber et al. 2015; Ayers et al. 2019; Sasayama et al. 2025). Previous studies have identified obstetric and sociodemographic predictors, including psychiatric history, primigravidity, cesarean delivery, and male infant birth (Liu et al. 2022; Yang et al. 2022). While psychiatric history remains a well-established psychological predictor, emerging evidence suggests that neurodevelopmental traits, specifically those associated with attention-deficit/hyperactivity disorder (ADHD) and autism spectrum disorder (ASD), may represent significant yet underrecognized vulnerability factors. Core features of ADHD (inattention, hyperactivity, and impulsivity) and ASD (communication and social difficulties, restricted interests, sensory atypicalities, and limited adaptability) may substantially hinder adaptation to pregnancy and parenting, thereby increasing the risk of depression.

An umbrella review by Kang et al. (2024) reported an odds ratio (OR) of 3.0 (95% confidence interval [CI] 1.53–5.92) for depression among individuals with ADHD traits. Similarly, a meta-analysis by Hudson et al. (2019) found that 48.6% of individuals with ASD experienced depression. In the perinatal context, women with ADHD face multiple risks during pregnancy and early parenting (Kittel-Schneider et al. 2021). Dorani et al. (2021) reported that 57.6% of women with ADHD experienced postpartum depression exceeding the rate in the general population. Likewise, qualitative studies have shown that women with ASD often struggle with communicative and sensory aspects of pregnancy and parenting (Gardner et al. 2016; Rogers et al. 2017). Hirokawa et al. (2019) conducted a large-scale study of Japanese women and used the Autism-Spectrum Quotient-10 (AQ-10) Japanese version to assess autistic traits, focusing on characteristics often described as part of the broader autism phenotype (BAP). They reported that women with such traits were at increased risk of postpartum depression and insecure maternal attachment at one month.

Taken together, these findings suggest that women with neurodevelopmental traits may have heightened vulnerability to perinatal depression, underscoring the importance of early screening and timely intervention. However, few studies have prospectively examined how such traits relate to depressive symptoms across the perinatal period.

In this manuscript, the term “perinatal depression” refers to depressive symptoms occurring during pregnancy and the early postpartum period. In the context of our study design, “perinatal” corresponds to the assessment window from late pregnancy to one month postpartum. Although DSM-5 defines postpartum depression as a major depressive episode with peripartum onset, in this study we distinguish between “antenatal depression” and “postpartum depression” based on the timing of assessment. Given that the study was not preregistered, the analyses were exploratory in nature.

Accordingly, this study examined how ADHD and ASD traits relate to depressive symptoms from late pregnancy through the early postpartum period, aiming to provide practical insights for more inclusive screening strategies and tailored mental health interventions in maternal care.

## Materials and methods

### Procedure and participants

A prospective longitudinal survey was conducted at four obstetric facilities in Nagano Prefecture, Japan between January 14 and December 31, 2020 (Clinical trial number: not applicable). Eligible participants were pregnant Japanese-speaking women aged 20 years or older who were able to complete the questionnaires. Gender identity was not assessed in this study. We therefore use the term “women” throughout this manuscript, consistent with the recruitment setting and the perinatal literature, while acknowledging that not all pregnant individuals identify as women.

At the 36–37 weeks antenatal checkup (T1), participants received the first survey set and provided written informed consent. Those who agreed completed questionnaires on neurodevelopmental traits, depressive symptoms, and sociodemographic characteristics. The second and third surveys were administered at the 2-week (T2) and 1-month postpartum checkups (T3), assessing depressive symptoms, childbirth experiences, and childcare. Neurodevelopmental traits were not reassessed postpartum as these traits are considered relatively stable over the short study period.

All data were anonymized and stored securely. Responses obtained at 14 ± 7 days postpartum were classified as T2, and those at 30 ± 7 days as T3. Questionnaires with missing data at a given timepoint were excluded from the corresponding analyses for that timepoint, while available data from other timepoints were retained.

### Measures

Three standardized self-report questionnaires were used to assess depressive symptoms and neurodevelopmental traits associated with ADHD and ASD.

#### Edinburgh postnatal depression scale

The Edinburgh Postnatal Depression Scale (EPDS) is a widely used 10-item screening tool designed to assess depressive symptoms during the perinatal period (Cox et al. 1987). The validated Japanese version (Okano et al. 1996) was used, with a cutoff of ≥ 9 indicating depressive risk. Although originally developed for postpartum use, later work has shown that the EPDS is applicable throughout the perinatal period, including antenatal assessments (Cox et al. 2014). Antenatal use has also been widely reported internationally (Evans et al. 2001; Limlomwongse and Liabsuetrakul 2006; Tendais et al. 2014). Because a validated antenatal cutoff has not been established in Japan, we applied the postpartum cutoff of ≥ 9 during pregnancy to ensure consistency across timepoints. Antenatal scores were interpreted cautiously, and analyses using continuous EPDS scores were also conducted.

#### Adult ADHD self-report scale

The Japanese version of the Adult ADHD Self-Report Scale v1.1 (ASRS) was used to assess ADHD traits (Takeda et al. 2017). The scale has 18 items with Part A containing six items most predictive of ADHD. Only Part A was analyzed. Participants with four or more positive responses were classified as ASRS-positive (ASRS+).

#### Autism spectrum quotient

The Autism Spectrum Quotient (AQ) is a self-report measure of autistic traits (Baron-Cohen et al. 2001). The validated Japanese version (Wakabayashi et al. 2004) was used. The scale includes 50 items across five domains: social skill, attention switching, attention to detail, communication, and imagination. A total score ≥ 33 was defined as AQ-positive (AQ+). For four domains with established cutoffs, scores above each threshold were coded as positive (Social Skills ≥ 6, Imagination ≥ 6, Attention Switching ≥ 7, Communication ≥ 6). The applicability of the AQ during pregnancy has been reported by Zain et al. (2023).

### Statistical analysis

Statistical analyses were conducted using SPSS Statistics software (version 29.0; IBM, Armonk, NY) and R (version 4.2.2). Descriptive statistics were calculated for mean EPDS scores and EPDS positivity at T1–T3. Spearman’s rank correlation assessed relationships among EPDS scores across timepoints. Cross-tabulation and chi-square tests were used to examine univariate associations between perinatal background factors and EPDS positivity, as well as neurodevelopmental characteristics (ASRS positivity and AQ positivity), with odds ratios calculated accordingly. Exploratory post hoc analyses were additionally performed for AQ subscales.

Multivariable logistic regression was conducted to identify independent predictors of EPDS positivity at T2. ASRS, AQ, and antenatal EPDS scores, all measured at T1, were entered as continuous variables, with primiparity, neonatal complications, and psychiatric treatment history as covariates. The term “predictor” refers to temporal precedence and does not imply causality.

Longitudinal changes in EPDS scores across T1–T3 were examined using a linear mixed-effects model (LMM). ASRS, AQ, time, and their interaction terms were included as fixed effects, with participant ID as a random intercept. Primiparity, neonatal complications, and psychiatric treatment history were included as covariates, as in the logistic regression model. Fixed-effect significance was evaluated using Type III ANOVA (Supplementary Table 7).

Because the analyses were exploratory, no correction for multiple testing was applied. Statistical significance was set at *p* < 0.05.

## Results

### Participants and characteristics

**Fig. 1:**
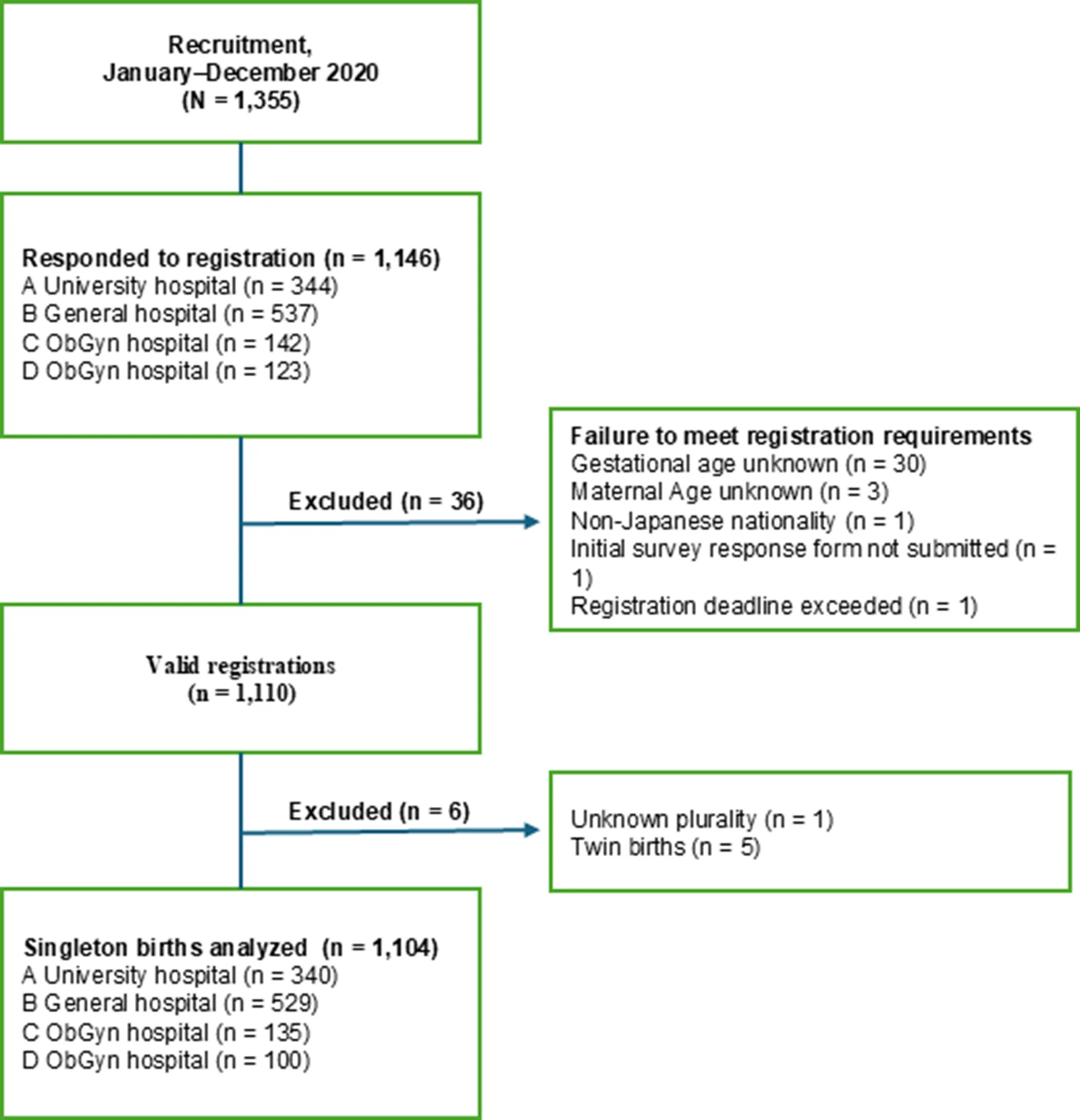
Flow of participant recruitment. Participants were recruited between January and December 2020. Only singleton births were included. Exclusion criteria were unknown gestational age, unknown maternal age, non-Japanese nationality, missing initial surveys, and registration after the deadline.

A total of 1,146 individuals participated (Fig. 1). After excluding 36 who did not meet eligibility criteria, 1,110 were enrolled. Data from 1,104 women with singleton live births were analyzed.

Table 1 summarizes participant characteristics. The mean age was 32.4 years (SD = 4.9) and mean gestational age at delivery was 39.6 weeks (SD = 1.1). Overall, 37.8% were primigravidas and 44.8% were primiparas. Most deliveries were vaginal (75.4%). A history of psychiatric treatment was reported by 138 women (12.5%).

**Table 1:** Obstetric demographics of participants

Variable	Range; Mean (SD)	n (%)
Age at registration, years	20–46; 32.4 (4.9)	
Pregnancy weeks at registration	36.0–37.9; 36.8 (0.6)	
Number of pregnancies^a^	1–5; 2.0 (1.1)	
Primigravida		417 (37.8%)
Multigravida		604 (54.7%)
Unknown		83 (7.5%)
Infertility treatment history		
Yes		245 (22.2%)
No		859 (77.8%)
Spontaneous abortion or still birth history		
Yes		190 (17.2%)
No		871 (78.9%)
Unknown		43 (3.9%)
Pregnancy complications		
Yes		224 (20.3%)
No		879 (79.6%)
Unknown		1 (0.1%)
Pregnancy weeks at delivery	36.4–42.1; 39.6 (1.1)	
Unknown		11 (1.0%)
Number of births^a^	1–5; 1.7 (0.8)	
Primipara		495 (44.8%)
Multipara		528 (47.8%)
Unknown		81 (7.3%)
Mode of delivery		
Cesarean		189 (17.1%)
Vaginal		832 (75.4%)
Unknown		83 (7.5%)
Baby sex		
Male		533 (48.3%)
Female		522 (47.3%)
Unknown		49 (4.4%)
Newborn weight, g	1,502–4,386; 3,077.4 (360.1)	
Unknown		25 (2.3%)
Newborn height, cm	40.3–56.0; 48.5 (1.8)	
Unknown		27 (2.5%)
Neonatal complications^a^		
Yes		168 (15.2%)
No		829 (75.1%)
Unknown		107 (9.7%)
Psychiatric treatment history		
Yes		138 (12.5%)
No		953 (86.3%)
Unknown		13 (1.2%)

*SD* standard deviation. *N* = 1,104. ^a^Reported at 2 weeks postpartum

Table 2 shows psychiatric diagnoses, with depression being the most common (*n* = 41). Six women had a prior ADHD diagnosis, and two had a previous ASD diagnosis. Screening identified 27 ASRS+ participants in the treatment-history group, although only three had a formal ADHD diagnosis. Seven were AQ+, although none had a formal ASD diagnosis.

**Table 2:** Distribution of psychiatric diagnoses in women with psychiatric treatment history, classified according to the ASRS+ and AQ+ screening results

	Women with psychiatric treatment history (*n* = 138)^a^	ASRS+ (*n* = 27)^b^	AQ+ (*n* = 7)
Diagnoses	n (%)	n (%)	n (%)
Depression	41 (29.7%)	9 (33.3%)	2 (28.6%)
Bipolar disorder	9 (6.5%)	3 (11.1%)	0
Panic disorder	17 (12.3%)	4 (14.8%)	0
Anxiety disorder	4 (2.9%)	0	0
Schizophrenia	7 (5.1%)	1 (3.7%)	0
OCD	6 (4.3%)	0	0
Eating disorders	2 (1.4%)	0	1 (14.3%)
Adjustment disorder	7 (5.1%)	1 (3.7%)	0
ADHD	6 (4.3%)	3 (11.1%)	0
ASD/PDD	2 (1.4%)	0	0
Others	24 (17.4%)	4 (14.8%)	2 (28.6%)
Unknown	21 (15.2%)	4 (14.8%)	1 (14.3%)
No response	7 (5.1%)	0	1 (14.3%)

ASRS + = ADHD traits positive on the Adult ADHD Self-Report Scale; AQ + = Autistic traits positive on the Autism-Spectrum Quotient; *OCD* obsessive–compulsive disorder, *PDD* pervasive developmental disorder. ^a^Fourteen women reported multiple diagnoses. ^b^Two women reported multiple diagnoses

### EPDS scores and correlations

**Fig. 2:**
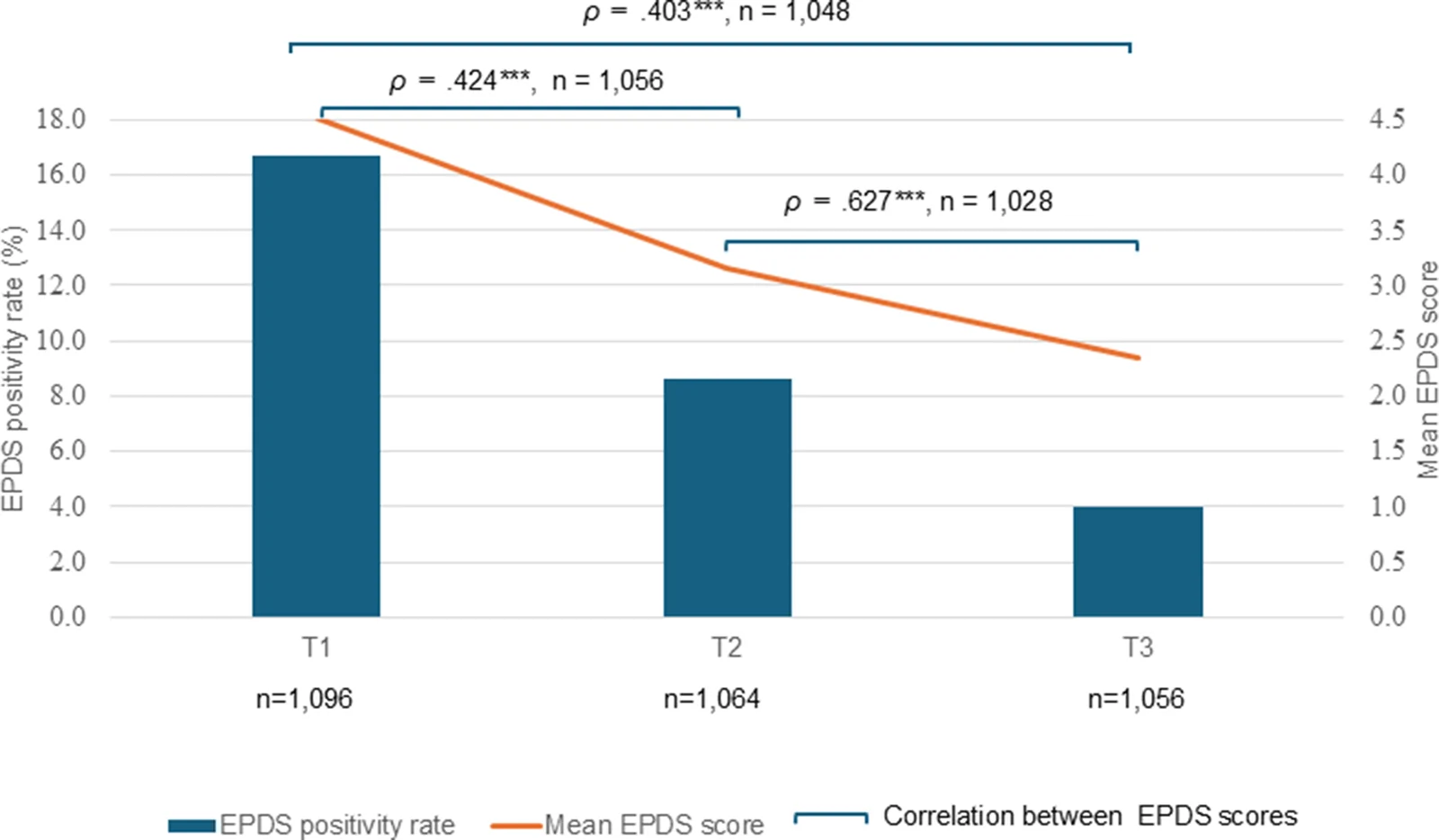
Mean EPDS scores, positivity rates, and correlations across the perinatal timepoints

Figure 2 shows mean EPDS scores and EPDS + at each timepoint. Depressive symptoms were most prevalent at T1 (16.7%), followed by T2 (8.6%) and T3 (4.0%). Spearman’s correlation analysis showed significant positive associations among EPDS scores across timepoints, with the strongest correlation between T2 and T3 (ρ = 0.627, *p* < 0.001).

### Associations between perinatal factors and EPDS positivity

Table 3 presents associations between perinatal factors and EPDS+. A history of psychiatric treatment was consistently associated with EPDS + at all three timepoints (T1: OR = 3.39, 95% CI 2.28–5.04, *p* < 0.001; T2: OR = 4.48, 95% CI 2.79–7.21, *p* < 0.001; T3: OR = 5.14, 95% CI 2.70–9.81, *p* < 0.001). At T1, aside from a history of psychiatric treatment, having a male fetus was the only significant factor (OR = 1.40, 95% CI 1.01–1.94, *p* < 0.05). At T2, primigravidity (OR = 4.01, 95% CI 2.49–6.46, *p* < 0.001) and primiparity (OR = 5.18, 95% CI 3.00–8.92, *p* < 0.001) were significant. At T3, primiparity remained significant, and neonatal complications also emerged as significant. No significant associations were found for infertility treatment, prior miscarriage or stillbirth, pregnancy complications, or mode of delivery.

**Table 3:** Associations between perinatal background factors and EPDS positivity across T1–3

	T1			T2			T3		
	EPDS ≥ 9	EPDS < 9		EPDS ≥ 9	EPDS < 9		EPDS ≥ 9	EPDS < 9	
	n (%)	n (%)	OR [95% CI]	n (%)	n (%)	OR [95% CI]	n (%)	n (%)	OR [95% CI]
Gravidity									
Primigravidity	72 (7.1)	341 (33.6)	1.06 [0.76–1.48]	63 (6.3)	346 (34.3)	4.01*** [2.49–6.46]	21 (2.1)	382 (38.7)	1.83 [0.95–3.52]
Multigravidity	100 (9.9)	501(49.4)		26 (2.6)	573 (56.8)		17 (1.7)	566 (57.4)	
Infertility treatment history									
Yes	33 (3.0)	211(19.3)	0.73 [0.49–1.10]	24 (2.3)	218 (20.5)	1.22 [0.75–1.99]	9 (0.9)	224 (21.2)	0.96 [0.45–2.04]
No	150 (13.7)	702 (64.1)		68 (6.4)	754 (70.9)		33 (3.1)	790 (74.8)	
Spontaneous abortion or stillbirth history									
Yes	35 (3.3)	154 (14.6)	1.16 [0.77–1.74]	13 (1.3)	172 (16.6)	0.75 [0.41–1.38]	7 (0.7)	177 (17.3)	0.97 [0.42–2.22]
No	142 (13.5)	722 (68.6)		78 (7.5)	774 (74.6)		33 (3.2)	808 (78.8)	
Pregnancy complications									
Yes	37 (3.4)	186 (17.0)	1.00 [0.67–1.48]	25 (2.4)	189 (17.8)	1.54 [0.95–2.51]	8 (0.8)	211 (20.0)	0.89 [0.41–1.96]
No	145 (13.2)	727 (66.4)		67 (6.3)	782 (73.6)		34 (3.2)	802 (76.0)	
Parity									
Primiparity	89 (8.8)	401 (39.5)	1.19 [0.85–1.65]	72 (7.1)	414 (41.0)	5.18*** [3.00–8.92]	29 (2.9)	449 (45.4)	3.60*** [1.68–7.68]
Multiparity	83 (8.2)	443 (43.6)		17 (1.7)	506 (50.1)		9 (0.9)	501 (50.7)	
Mode of delivery									
Cesarean	33 (3.3)	156 (15.4)	1.04 [0.69–1.59]	12 (1.2)	176 (17.5)	0.66 [0.35–1.23]	4 (0.4)	175 (17.7)	0.52 [0.18–1.48]
Vaginal	139 (13.7)	686 (67.7)		77 (7.6)	742 (73.7)		34 (3.4)	773 (78.4)	
Baby sex									
Male	101(9.6)	427 (40.8)	1.40* [1.01–1.94]	47 (4.5)	473 (45.7)	1.04 [0.68–1.60]	19 (1.9)	492 (48.3)	0.94 [0.50–1.78]
Female	75 (7.2)	444 (42.4)		45 (4.3)	471 (45.5)		20 (2.0)	487 (47.8)	
Neonatal complications									
Yes	31 (3.1)	135 (13.6)	1.17 [0.76–1.81]	19 (1.9)	149 (15.0)	1.42 [0.83–2.43]	12 (1.2)	144 (14.9)	2.61** [1.28–5.32]
No	135 (13.6)	689 (69.6)		68 (6.9)	755 (76.2)		25 (2.6)	784 (81.2)	
Psychiatric treatment history									
Yes	48 (4.4)	89 (8.2)	3.39*** [2.28–5.04]	32 (3.0)	102 (9.7)	4.48*** [2.79–7.21]	17(1.6)	117(11.2)	5.14*** [2.70–9.81]
No	130 (12.0)	817 (75.4)		60 (5.7)	857(81.5)		25(2.4)	885(84.8)	

*p < 0.05, **p < 0.01, ***p < 0.001

T1 = antenatal assessment at 36–37 weeks of gestation; T2 = 2-week postpartum (14 ± 7 days); T3 = 1-month postpartum (30 ± 7 days)

*OR* odds ratio, *CI* confidence interval

### Associations between neurodevelopmental traits and EPDS positivity

Table 4 shows associations between neurodevelopmental traits and EPDS+. Individuals who screened ASRS + had higher odds of EPDS + at all three timepoints (T1: OR = 4.86, 95% CI 3.06–7.72, *p* < 0.001; T2: OR = 3.65, 95% CI 2.07–6.42, *p* < 0.001; T3: OR = 4.23, 95% CI 2.00–8.96, *p* < 0.001). In contrast, those who screened AQ+ showed elevated risk only at T1 (OR = 6.08, 95% CI 2.17–16.99, *p* < 0.001), with no significant associations at T2 or T3. Post hoc domain-specific analyses showed that higher scores on the social skill, attention switching, and communication subscales were associated with EPDS + at all timepoints. The imagination subscale showed no significant associations. Four participants exceeded both ASRS + and AQ+ cutoffs. Their mean EPDS scores were 8.75 at T1, 5.75 at T2, and 3.50 at T3, with EPDS+ rates of 75%, 25%, and 0%, respectively. Because this subgroup was extremely small, only descriptive results were reported.

**Table 4:** Associations between neurodevelopmental characteristics and EPDS positivity across T1–3

	T1			T2			T3		
	EPDS ≥ 9	EPDS < 9		EPDS ≥ 9	EPDS < 9		EPDS ≥ 9	EPDS < 9	
	n (%)	n (%)	OR [95% CI]	n (%)	n (%)	OR [95% CI]	n (%)	n (%)	OR [95% CI]
ASRS score									
≥ 4	38 (3.5%)	47 (4.3%)	4.86*** [3.06–7.72]	19 (1.8%)	64 (6.1%)	3.65*** [2.07–6.42]	10 (1.0%)	69 (6.6%)	4.23*** [2.00–8.96]
< 4	143 (13.1%)	860 (79.0%)		73 (6.9%)	897 (85.2%)		32 (3.1%)	934 (89.4%)	
AQ total score									
≥ 33	8 (0.8%)	7 (0.7%)	6.08*** [2.17–16.99]	2 (0.2%)	14 (1.4%)	1.54 [0.34–6.89]	1 (0.1%)	14 (1.4%)	1.75 [0.23–13.65]
< 33	164 (15.6%)	872 (83.0%)		85 (8.4%)	916 (90.1%)		39 (3.9%)	956 (94.7%)	
AQ subscale score									
Social skill									
≥ 6	86 (8.2%)	245 (23.3%)	2.59*** [1.85–3.61]	37 (3.6%)	283 (27.8%)	1.69* [1.08–2.65]	22 (2.2%)	296 (29.3%)	2.78** [1.47–5.27]
< 6	86 (8.2%)	634 (60.3%)		50 (4.9%)	647 (63.6%)		18 (1.8%)	674 (66.7%)	
Attention switching									
≥ 7	38 (3.6%)	51 (4.9%)	4.60*** [2.91–7.28]	18 (1.8%)	73 (7.2%)	3.06*** [1.73–5.42]	8 (0.8%)	80 (7.9%)	2.78* [1.24–6.24]
< 7	134 (12.7%)	828 (78.8%)		69 (6.8%)	857 (84.3%)		32 (3.2%)	890 (88.1%)	
Communication									
≥ 6	31 (2.9%)	55 (5.2%)	3.29*** [2.05–5.30]	13 (1.3%)	72 (7.1%)	2.09* [1.11–3.96]	10 (1.0%)	74 (7.3%)	4.04*** [1.90–8.58]
< 6	141 (13.4%)	824 (78.4%)		74 (7.3%)	858 (84.4%)		30 (3.0%)	896 (88.7%)	
Imagination									
≥ 6	20 (1.9%)	67 (6.4%)	1.60 [0.94–2.71]	9 (0.9%)	77 (7.6%)	1.28 [0.62–2.65]	5 (0.5%)	78 (7.7%)	1.63 [0.62–4.29]
< 6	152 (14.5%)	812 (77.3%)		78 (7.7%)	853 (83.9%)		35 (3.5%)	892 (88.3%)	

*p < 0.05, **p < 0.01, ***p < 0.001

T1 = antenatal assessment at 36–37 weeks of gestation; T2 = 2-week postpartum (14 ± 7 days); T3 = 1-month postpartum (30 ± 7 days)

*OR* odds ratio, *CI* confidence interval

### Predictors of postpartum depression

Multivariable logistic regression analyses were conducted to examine the independent contribution of neurodevelopmental traits to postpartum depressive symptoms (Table 5). The outcome variable was EPDS + at T2. This timepoint was selected because EPDS + was higher at T2 than at T3, and because T2 closely reflected early postpartum mental health, as shown in Fig. 2. ASRS and AQ total scores were entered as continuous predictors.

**Table 5:** Logistic regression models examining associations with EPDS positivity at 2 weeks postpartum (T2)

Model	Association variable	Coefficient (B)	SE	OR [95% CI]
Model 1 (*n* = 1,009)				
	ASRS total score	0.44	0.08	1.54*** [1.32–1.80]
	AQ total score	0.05	0.02	1.05* [1.01–1.08]
	Constant	-3.90	0.36	
Model 2 (*n* = 1,006)				
	Antenatal EPDS score	0.18	0.03	1.19*** [1.14–1.26]
	ASRS total score	0.29	0.09	1.34*** [1.14–1.58]
	AQ total score	0.01	0.02	1.01 [0.98–1.05]
	Constant	-3.79	0.37	
Model 3 (*n* = 929)				
	Antenatal EPDS score	0.16	0.03	1.17*** [1.11–1.24]
	ASRS total score	0.31	0.10	1.37** [1.13–1.66]
	AQ total score	0.00	0.02	1.00 [0.96–1.04]
	Primiparity	2.01	0.33	7.47*** [3.88–14.38]
	Neonatal complication	0.55	0.31	1.74 [0.94–3.21]
	Psychiatric treatment history	1.12	0.32	3.08*** [1.66–5.72]
	Constant	-5.08	0.50	

*p < 0.05, **p < 0.01, ***p < 0.001. *SE* standard errors, *OR* odds ratio, *CI* confidence interval

Model 1 only includes the ASRS and AQ total scores, with no covariates. Model 2 includes the ASRS total score, AQ total score, and antenatal EPDS score. Model 3 includes the ASRS total score, AQ total score, antenatal EPDS score, primiparity, neonatal complications, and psychiatric treatment history. T2 = 2 weeks postpartum (14 ± 7 days)

In Model 1 (unadjusted), higher ASRS (*p* < 0.001) and AQ (*p* = 0.012) scores were associated with EPDS+. In Model 2, which included antenatal EPDS scores as a continuous covariate, ASRS remained significant (*p* < 0.001), whereas AQ was not (*p* = 0.453). Antenatal EPDS scores were also associated with EPDS+ (*p* < 0.001).

In Model 3, which additionally included antenatal EPDS scores and perinatal covariates, ASRS remained an independent predictor (OR = 1.37, 95% CI 1.13–1.66, *p* < 0.01), while AQ remained nonsignificant (OR = 1.00, 95% CI 0.96–1.04, *p* = 0.962). Primiparity, psychiatric treatment history, and antenatal EPDS scores were also significant predictors, whereas neonatal complications were not (Table 5).

### Longitudinal changes in EPDS scores

To complement these analyses, a linear mixed-effects model (LMM) was conducted to examine longitudinal changes in EPDS scores across T1–T3 (Table 6). EPDS scores were treated as repeated continuous outcomes.

**Table 6:** Linear mixed model estimates for EPDS scores (Interaction model)

Variable	B	SE	95% CI	*p*
(Intercept)	1.46	0.32	[0.83, 2.08]	< 0.001
Timepoint (Ref: T1)				
T2	0.31	0.34	[-0.35, 0.98]	0.36
T3	-0.11	0.34	[-0.77, 0.56]	0.76
Neurodevelopmental traits				
ASRS	0.81	0.09	[0.64, 0.98]	< 0.001
AQ	0.13	0.02	[0.10, 0.16]	< 0.001
Covariates				
Parity (Ref: Multiparity)				
Primiparity	1.27	0.16	[0.95, 1.59]	< 0.001
Neonatal complication (Ref: Absent)				
Present	0.73	0.22	[0.30, 1.16]	0.001
Psychiatric history (Ref: Absent)				
Present	1.74	0.25	[1.24, 2.24]	< 0.001
Interaction effects				
Time (T2) × ASRS	-0.31	0.09	[-0.49, -0.12]	0.001
Time (T3) × ASRS	-0.55	0.10	[-0.74, -0.37]	< 0.001
Time (T2) × AQ	-0.07	0.02	[-0.11, -0.03]	< 0.001
Time (T3) × AQ	-0.08	0.02	[-0.11, -0.04]	< 0.001

Random effects

Subject intercept (τ00): Variance = 4.10, SD = 2.03

Residual (σ²): Variance = 6.46, SD = 2.54

Model information

REML criterion = 14161.1

Observations = 2793

Subjects = 938

*EPDS* Edinburgh postnatal depression scale, *ASRS* Adult ADHD self-report scale; *AQ* Autism-spectrum quotient, T1 antenatal assessment at 36–37 weeks of gestation; T2 = 2-week postpartum (14 ± 7 days); T3 = 1-month postpartum (30 ± 7 days); Main effects of ASRS and AQ represent associations at T1 (reference timepoint)

The intercept was 1.46 (SE = 0.32, *p* < 0.001). The main effect of time was not significant (F (2, 1854.79) = 0.82, *p* = 0.439). Higher ASRS scores were associated with elevated EPDS scores at T1 (B = 0.81, SE = 0.09, *p* < 0.001). AQ scores also showed a positive association at T1 (B = 0.13, SE = 0.02, *p* < 0.001).

Significant interaction effects were observed for ASRS×Time, indicating attenuation of the ASRS–EPDS association at later timepoints (T2: B = − 0.31, SE = 0.09, *p* = 0.001; T3: B = − 0.55, SE = 0.10, *p* < 0.001). A similar pattern was observed for AQ×Time (T2: B = − 0.07, SE = 0.02, *p* < 0.001; T3: B = − 0.08, SE = 0.02, *p* < 0.001). Regarding covariates, primiparity was associated with higher EPDS scores (B = 1.27, SE = 0.16, *p* < 0.001). Neonatal complications (B = 0.73, SE = 0.22, *p* = 0.001) and psychiatric treatment history (B = 1.74, SE = 0.25, *p* < 0.001) were also associated with higher EPDS scores.

## Discussion

EPDS scores and the proportion of EPDS+ were highest at T1 and declined by T3. The postpartum prevalence of EPDS + was lower than the commonly reported 10–20% in perinatal populations, likely due to contextual factors. Japan has strengthened national policies to prevent postpartum depression, promoting early detection and intervention in obstetric and municipal settings. Additionally, many women receive supportive care at their parental home, which may buffer distress.

EPDS scores across T1–T3 were significantly correlated, suggesting that identifying elevated symptoms during pregnancy may enable early intervention.

Although AQ + was not associated with postpartum EPDS positivity, continuous AQ scores were associated with EPDS + at T2 before adjustment for antenatal EPDS scores, and three of four AQ subscales were associated with EPDS + at all timepoints. These findings are broadly consistent with Hirokawa et al. (2019), who reported that BAP, assessed using the AQ-10 (10 items; cutoff ≥ 7), was associated with postpartum depressive symptoms. The discrepancy for AQ + may reflect methodological differences: the stringent AQ cutoff (≥ 33) identified few women, limiting statistical power, whereas the AQ-10 captures milder BAP-related traits.

Multivariable logistic regression further showed that antenatal EPDS scores, ASRS scores, primiparity, and psychiatric treatment history independently predicted EPDS + at T2. In contrast, AQ total scores did not emerge as an independent predictor, suggesting that the association between AQ scores and postpartum symptoms was largely explained by antenatal depressive symptoms.

Longitudinally, the non-significant main effect of time reflected stable, low EPDS scores at the model reference point (ASRS = AQ = 0). In contrast, the significant interactions between time and ASRS, as well as time and AQ, indicated that higher neurodevelopmental trait scores were associated with higher antenatal EPDS scores and larger postpartum decreases. Thus, antenatal depressive symptoms related to neurodevelopmental traits tended to subside after delivery; however, regression to the mean and the concurrent assessment of traits and symptoms at T1 may partly explain this pattern. Although these associations were attenuated postpartum, women with high trait scores may benefit from continued monitoring.

Taken together, this study highlights that, beyond established risk factors such as psychiatric treatment history and primiparity, neurodevelopmental characteristics assessed during pregnancy warrant attention. Some women without a documented psychiatric history may nonetheless exhibit neurodevelopmental traits relevant to perinatal mental health. In perinatal care settings, relying solely on diagnoses may overlook them. Brief psychological assessment during antenatal visits may support early identification of maternal characteristics and enable tailored, reasonable accommodations.

## Limitations

This study has several limitations. First, neurodevelopmental traits were assessed using self-report screening tools, which may be subject to reporting biases. Second, the generalizability of the findings is limited. Participants were women attending routine antenatal checkups at general obstetric outpatient clinics and were relatively stable physically and psychologically; therefore, individuals requiring intensive medical or psychiatric care were not included. Such excluded groups may experience higher psychological distress, and depressive symptoms may have been underestimated in the present sample. In addition, all participants were Japanese women, and cultural or institutional factors specific to Japan—such as patterns of social support, expectations surrounding motherhood, and maternity care practices—may have influenced the associations.

Third, although ASD subdomains showed differential associations with depressive symptoms, domain-level analyses using logistic regression or linear mixed-effects models could not be conducted. For ADHD traits, domain-specific analyses of inattention and hyperactivity were also not performed. More fine-grained analyses may help clarify which specific neurodevelopmental characteristics, beyond diagnostic categories, are most strongly related to perinatal depressive symptoms.

Fourth, the longitudinal design included only three assessment points with relatively short intervals, which may have been insufficient to capture nonlinear changes or longer-term trajectories. Depressive symptoms beyond 1 month postpartum were not assessed, and the relatively small number of participants with elevated AQ scores may have limited the precision and statistical power of estimates related to autistic traits. Therefore, the non-significant associations for AQ+ should not be interpreted as evidence of no association.

Finally, psychosocial variables such as social support, partner relationships, and economic status were not statistically adjusted, and potential interactions between these factors and neurodevelopmental traits were not captured. Moreover, the study was not preregistered, and replication in independent samples is needed to confirm these findings.

## Conclusions

This study demonstrated that maternal neurodevelopmental traits were associated with depressive symptoms from pregnancy through the early postpartum period, underscoring the relevance of a neurodiversity-informed perspective in perinatal mental health. Incorporating dimensional assessments of neurodevelopmental traits into perinatal care and providing support tailored to each mother’s trait profile may help promote psychological stability and facilitate adaptive caregiving across pregnancy and the postpartum period.

## Supplementary Information

Below is the link to the electronic supplementary material.

Supplementary Material 1

## Data Availability

The datasets generated and analyzed during the current study are not publicly available due to the sensitive nature of the clinical data and the risk of participant re-identification.
