# Supplementary material for: Associations between neurodevelopmental traits and perinatal depression: a prospective study in Japanese women
Source: Arch Womens Ment Health. 2026 Nov 4;29(6):146. doi: 10.1007/s00737-026-01781-4 (PMC13736413; doi:10.1007/s00737-026-01781-4)
Supplement: Supplementary file 1 [file 737_2026_1781_MOESM1_ESM.docx]

**Supplementary Table 7 Type III Analysis of Variance (ANOVA) for the Linear Mixed Model**

| **Effect** | **NumDF** | **DenDF** | **F** | **p** |  |
| --- | --- | --- | --- | --- | --- |
| **ASRS** | 1 | 931.79 | 62.65 | < .001 |  |
| **Time** | 2 | 1854.79 | 0.82 | 0.439 |  |
| **AQ** | 1 | 929.51 | 36.48 | < .001 |  |
| **Primiparity** | 1 | 932.25 | 60.04 | < .001 |  |
| **Neonatal Complications** | 1 | 932.68 | 10.97 | < .001 |  |
| **Psychiatric history** | 1 | 930.57 | 47.03 | < .001 |  |
| **Time × ASRS** | 2 | 1855.46 | 16.98 | < .001 |  |
| **Time × AQ** | 2 | 1853.02 | 9.81 | < .001 |  |
| NumDF = numerator degrees of freedom; DenDF = denominator degrees of freedom (Satterthwaite approximation). | | | | |  |
